# Synthesis, cytotoxic assessment, and molecular docking studies of 2,6-diaryl-substituted pyridine and 3,4- dihydropyrimidine-2(1H)-one scaffolds

**DOI:** 10.3906/kim-1903-72

**Published:** 2020-02-11

**Authors:** Zahra HOSSEINZADEH, Nima RAZZAGHI-ASL, Ali RAMAZANI, Hamideh AGHAHOSSEINI, Ali RAMAZANI

**Affiliations:** 1 Department of Chemistry, University of Zanjan, Zanjan Iran; 2 Department of Medicinal Chemistry, School of Pharmacy, Ardabil University of Medical Sciences, Ardabil Iran; 3 Cancer Gene Therapy Research Center, Zanjan University of Medical Sciences, Zanjan Iran; 4 Research Institute of Modern Biological Techniques, University of Zanjan, Zanjan Iran

**Keywords:** Cancer, cytotoxicity, kinesin Eg5, pyrimidinone, pyridine, magnetic nanocatalyst

## Abstract

Cancer is one of the main global health problems. In order to develop novel antitumor agents, we synthesized 3,4-dihydropyrimidine-2(1H)-one (DHPM) and 2,6-diaryl-substituted pyridine derivatives as potential antitumor structures and evaluated their cytotoxic effects against several cancer cell lines. An easy and convenient method is reported for the synthesis of these derivatives, employing cobalt ferrite (CoFe _2_ O _4_ @SiO _2_ -SO _3_ H) magnetic nanoparticles under microwave irradiation and solvent-free conditions. The structural characteristics of the prepared nanocatalyst were investigated by FTIR, XRD, SEM, and TGA techniques. In vitro cytotoxic effects of the synthesized products were assessed against the human breast adenocarcinoma cell line (MCF-7), gastric adenocarcinoma (AGS), and human embryonic kidney (HEK293) cells via MTT assay. The results indicated that compound **4r** (DHPM derivative) was the most toxic molecule against the MCF-7 cell line (IC _50_ of 0.17 μg/mL). Moreover, compounds **4j** and **4r** (DHPM derivatives) showed excellent cytotoxic activities against the AGS cell line, with an IC _50_ of 4.90 and 4.97 μg/mL, respectively. Although they are pyridine derivatives, compounds **5g** and **5m** were more active against the MCF-7 cell line. Results showed that the candidate compounds exhibited low cytotoxicity against HEK293 cells. The kinesin Eg5 inhibitory potential of the candidate compounds was evaluated by molecular docking. The docking results showed that, among the pyridine derivatives, compound **5m** had the most free energy of binding (–9.52 kcal/mol) and lowest Ki (0.105 μM), and among the pyrimidine derivatives, compound **4r** had the most free energy of binding (–7.67 kcal/mol) and lowest Ki (2.39 μM). Ligand-enzyme affinity maps showed that compounds **4r** and **5m** had the potential to interact with the Eg5 binding site via H-bond interactions to GLU116 and GLY117 residues. The results of our study strongly suggest that DHPM and pyridine derivatives inhibit important tumorigenic features of breast and gastric cancer cells. Our results may be helpful in the further design of DHPMs and pyridine derivatives as potential anticancer agents.

## 1. Introduction

A common approach in cancer chemotherapy is the development of drugs that interrupt the mitosis phase of cell division. The mitotic spindle is an important target in cancer chemotherapy, and a fundamental spindle motor protein is kinesin Eg5, which is considered as an important therapeutic target due to its specific role during mitosis in assembly [1,2].

In recent years, 3,4-dihydropyrimidine-2(1H)-ones (DHPMs) have been identified as compounds that specifically inhibit Eg5 function. Since mitotic kinesins are exclusively involved in the formation of the mitotic spindle, the inhibition of Eg5 by DHPMs is considered an attractive approach to cancer treatment [3]. It was reported that monastrol has an antitumor effect on various cancer cell types, such as breast, renal, and glioma cell lines. Unlike taxol, this compound, as an antimitotic agent, has not showed neuronal cytotoxicity [4,5].

Recently, DHPMs have played a significant role in medicinal chemistry due to their wide range of biological activities, such as antitumor [6], antiinflammatory [7], antihypertensive [8], and antiviral [9] activities, and, most importantly, as calcium channel modulators [10]. Moreover, pyridine derivatives are known to have multiple biological effects, such as anticancer [11], anti-HIV virus [12], antimicrobial [13], antiin?ammatory [14] cardiotonic [15], and antiparkinsonism properties [16]. Several 4,6-diaryl-substituted and tricyclic 2-amino-3-cyanopyridines have shown antitumor effects on human breast cancer cell lines T-47D and ZR-75-1 [17,18].

Most cancer drugs usually show high toxicity and/or multidrug resistance of tumors. Hence, the development of novel bioactive compounds with anticancer effects is a very important issue [17,18]. Much research has been conducted on pyrimidine and pyridine derivatives; however, the simple synthesis and cytotoxic activity profiles of 2,6-diaryl-substituted pyridine and DHPMs on the human breast adenocarcinoma cell line (MCF-7), gastric adenocarcinoma (AGS), and human embryonic kidney (HEK293) cells has not been explored thus far. This paper reports the simple and efficient synthesis, cytotoxic assessment, and molecular docking studies of 2,6-diaryl-substituted pyridine and DHPM derivative scaffolds.

In light of the importance of DHPM derivatives, several improved methods have been reported for the preparation of these compounds using various catalysts [19–22]. Although multiple methods have been reported for preparing functionalized pyridine derivatives [23–26], most require multiple steps, a large excess of expensive reagents, long reaction times, toxic solvents, and forceful reaction conditions.

**Scheme 1:**
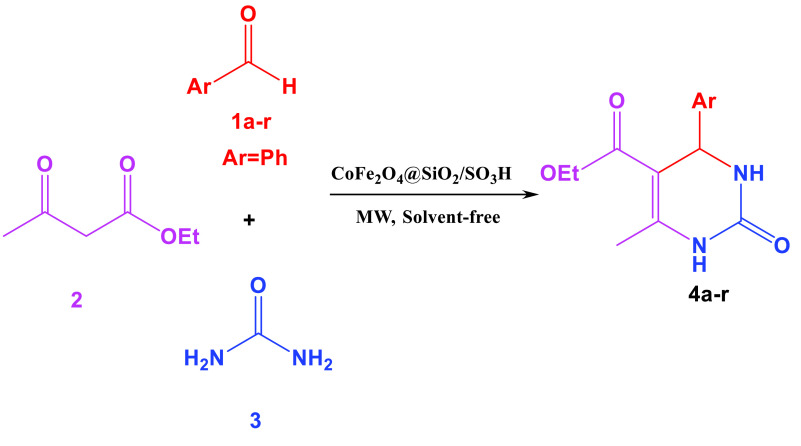
Synthesis of DHPMs in the presence of the CoFe _2_ O _4_ @SiO _2_ -SO _3_ H MNPs.

**Scheme 2:**
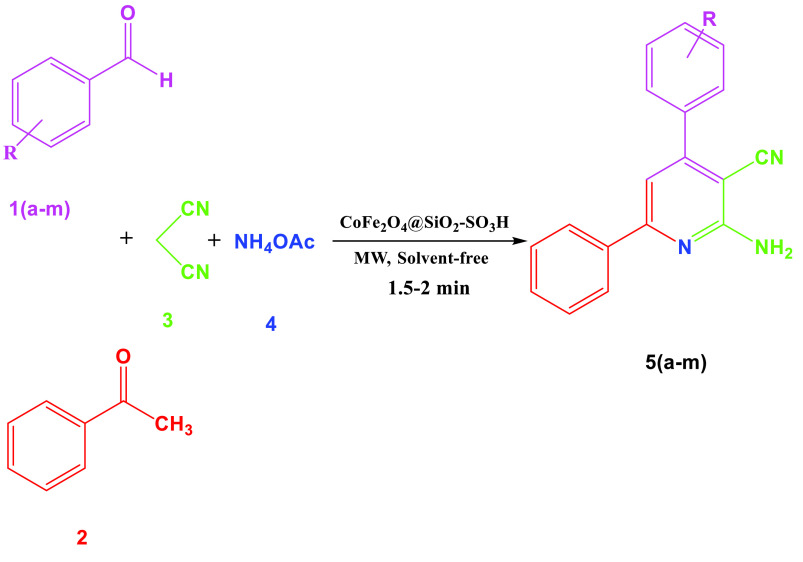
Synthesis of 2-amino-4,6-diarylnicotinonitriles in the presence of CoFe _2_ O _4_ @SiO _2_ -SO _3_ H MNPs.

In view of these reports, and in continuation of our previous work towards the development of efficient and environmentally benign heterogeneous catalysts [27–30], we synthesized some DHPM/pyridine derivatives in the presence SO3 H-substituted silica-coated cobalt-based (CoFe _2_ O _4_ @SiO _2_ -SO _3_ H) magnetic nanoparticles (MNPs) as a highly efficient catalyst under solvent-free conditions with microwave irradiation (Schemes 1 and 2) to evaluate their cytotoxic effects on MCF-7, AGS, and HEK293 cells. The CoFe _2_ O _4_ @SiO _2_ -SO _3_ H MNPs were synthesized as highly efficient magnetic nanocatalysts and employed for the synthesis of DHPM and pyridine derivatives under solvent-free conditions with microwave irradiation (Schemes 1 and 2). Finally, the screened derivatives were subjected to molecular docking simulations to explore their binding potential toward the Eg5 enzyme as a validated cancer target for these compounds.

## 2. Results and discussion

### 2.1. Catalyst characterization

An easy and efficient procedure was employed for the preparation of the CoFe _2_ O _4_ @SiO _2_ -SO _3_ H core-shell composite with CoFe _2_ O _4_ spheres as the core and SiO _2_ -SO _3_ H MNPs as the shell. There were a great number of hydroxyl groups on the surface of the CoFe _2_ O _4_ @SiO _2_ -SO _3_ H MNPs. The MNPs were simply coated with amorphous SiO _2_ using the conventional sol-gel procedure and then functionalized by SO _3_ H groups via simple mixing with ClSO _3_ H.

**Figure 1:**
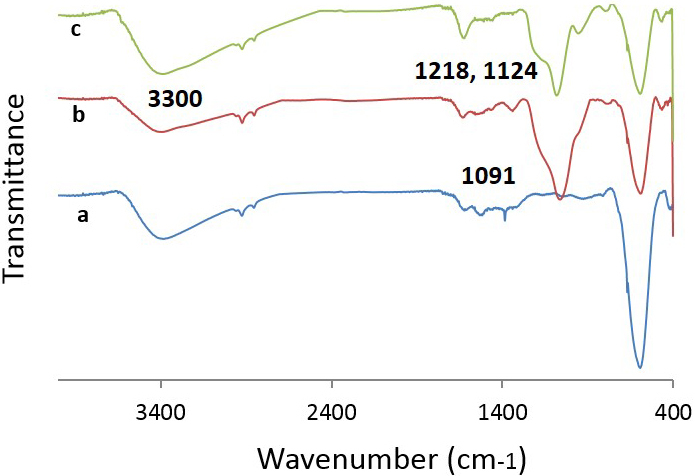
FTIR spectra of (a) CoFe _2_ O _4_ , (b) CoFe _2_ O _4_ @SiO _2_ , and (c) CoFe _2_ O _4_ @SiO _2_ -SO _3_ H MNPs.

The structural characteristics of the CoFe _2_ O _4_ @SiO _2_ -SO _3_ H MNPs were studied via TGA, SEM, FTIR, and XRD techniques. The FTIR spectra of the CoFe _2_ O _4_ , CoFe _2_ O _4_ @SiO _2_ , and CoFe _2_ O _4_ @SiO _2_ -SO _3_ H are represented in Figure 1. Two characteristic bands at 3300 and 592 cm ^-1^ were related to the O-H stretching of the sulfonic acid functional group and M-O vibration, respectively [31]. The bands that appeared at 1218 and 1124 cm ^-1^ belonged to the sulfonyl group that overlapped with a stronger peak of Si-O at 1091 cm ^-1^ . The peaks at 1061, 1073, and 474 cm ^-1^ confirmed the presence of Si–O–Si moiety in the nanocatalyst structure [32,33]. The presence of peaks at ranges of 592–629 cm ^-1^ and 1091–1218 cm ^-1^ can be considered as similarities of the 3 stages (a, b, and c) and 2 stages (b and c), respectively. The peaks that appeared at 1218 and 1124 cm ^-1^ , which overlapped with the stronger peak of Si-O at 1091 cm ^-1^ , were the differences of stages a and c.

**Figure 2:**
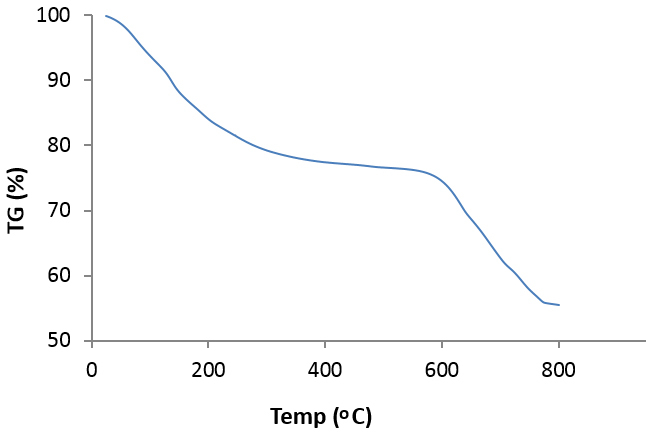
TGA curves of CoFe _2_ O _4_ @SiO _2_ -SO _3_ H MNPs.

The TGA curve of CoFe _2_ O _4_ @SiO _2_ -SO _3_ H MNPs demonstrated the weight losses of the surface hydroxyl groups and physically adsorbed solvent below 150 °C (Figure 2). Moreover, weight loss in the range of 600–800 °C was attributed to the weight loss of the SO _3_ H groups in the nanocatalyst structure [34].

**Figure 3:**
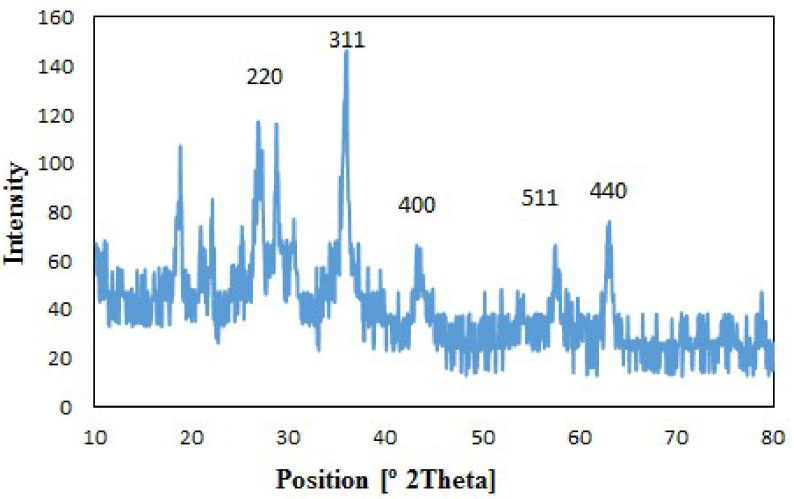
XRD pattern of CoFe _2_ O _4_ @SiO _2_ -SO _3_ H MNPs.

The crystallinity and the average diameter of the CoFe _2_ O _4_ @SiO _2_ -SO _3_ H MNPs were obtained from the analysis of the XRD pattern. The XRD pattern of the CoFe _2_ O _4_ @SiO _2_ -SO _3_ H MNPs is presented in Figure 3, where it can be seen that the CoFe _2_ O _4_ @SiO _2_ -SO _3_ H MNPs exhibited 5 characteristic bands at the 2θ values of (220), (311), (400), (511), and (440), corresponding to a cubic spinal structure of CoFe _2_ O _4_ (card no. 00-001-1121) [35]. The diameter of the CoFe _2_ O _4_ @SiO _2_ -SO _3_ H MNPs was measured by XRD employing the Debye-Scherrer equation at about 33 nm.

**Figure 4:**
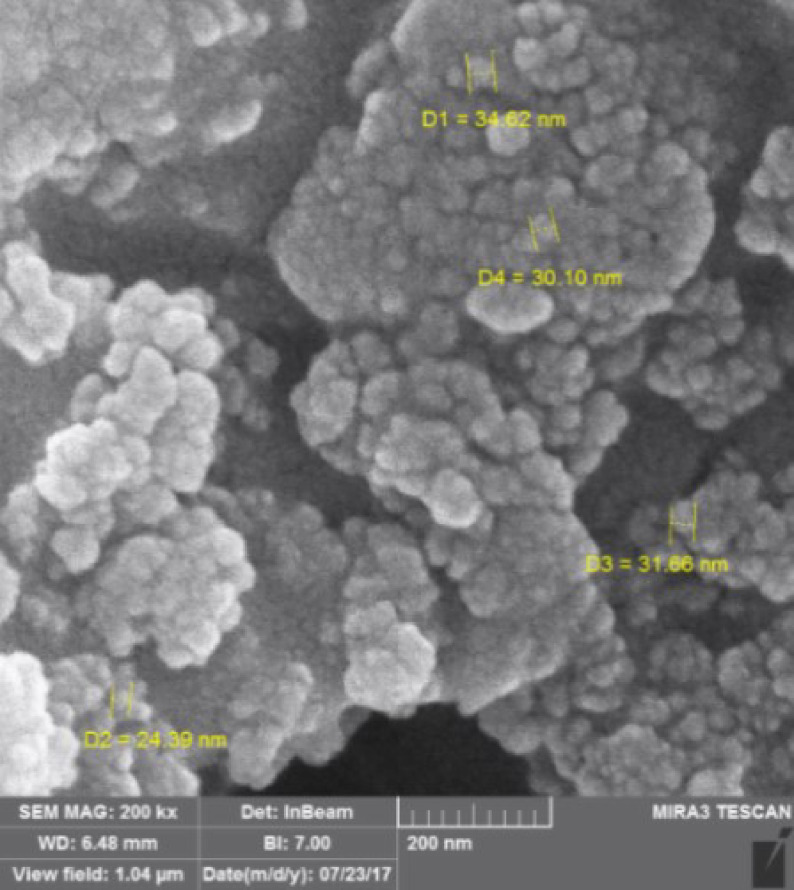
SEM image of CoFe _2_ O _4_ @SiO _2_ -SO _3_ H MNPs.

The SEM analysis revealed that the CoFe _2_ O _4_ @SiO _2_ -SO _3_ H MNPs had a nanocrystalline structure with nanodimensions ranging from 22.98 to 45.30 nm (Figure 4).

### 2.2. Catalytic effect of CoFe _2_ O _4_ @SiO _2_ -SO _3_ H for the synthesis of DHPM and 2-amino-4,6-diarylnicotinonitrile derivatives

After characterization of the catalyst, its catalytic effect on the synthesis of DHPMs was evaluated. To optimize the reaction conditions, the reaction of 4-chlorobenzaldehyde, ethyl acetoacetate, and urea was evaluated under different microwave powers in the presence of various amounts of CoFe _2_ O _4_ @SiO _2_ -SO _3_ H as a model reaction (Tables 1 and 2). In the absence of a nanocatalyst, only trace amounts of the products could be obtained (Table 1).

**Table 1:** Effect of the CoFe _2_ O _4_ @SiO _2_ -SO _3_ H nanocatalyst amount on the synthesis of DHPMs.

Entry	Catalyst (g)	Time (min)	Yield (%)
1	None	8	Trace
2	0.010	8	68
3	0.015	3	90
4	0.020	3	79

Reaction situations: 4-Chlorobenzaldehyde 1 (1 mmol), urea 2 (2 mmol), and ethyl acetoacetate 3 (1.5 mmol) under solvent-free conditions with microwave irradiation at 400 W.

**Table 2:** Effect of microwave power on the synthesis of DHPMs in the presence of CoFe _2_ O _4_ @SiO _2_ -SO _3_ H.

Entry	Microwave power (W)	Time (min)	Yield (%)
1	300	3	63
2	350	3	72
3	400	3	90
4	450	3	80

Reaction conditions: 4-Chlorobenzaldehyde 1 (1 mmol), urea 2 (2 mmol), ethyl acetoacetate 3 (1.5 mmol), and CoFe2O4@SiO _2_ -SO3H (0.015 g), with microwave irradiation under solvent-free conditions.

After reaction optimization, the reactions of ethyl acetoacetate with different aldehydes (1a–1r) and urea were performed in the presence of CoFe _2_ O _4_ @SiO _2_ -SO _3_ H MNPs under microwave irradiation within 3–5 min (Table 3).

**Table 3:** Synthesis of DHPM derivatives by 3-component condensation of aldehydes, urea, and ethyl acetoacetate in the presence of CoFe _2_ O _4_ @SiO _2_ -SO _3_ H.

No.	Ar	Product	Time (min)	Yield (%)	Mp (°C) Ref.
1	Ph	4a	5	90	198–201 [37]
2	4-Me-Ph	4b	5	86	206–209 [38]
3	4-Cl-Ph	4c	3	90	212–215 [37]
4	3-Cl-Ph	4d	5	92	192–194 [39]
5	2-Cl-Ph	4e	5	89	210–213 [37]
6	4-Br-Ph	4f	3	86	211–214 [40]
7	3-Br-Ph	4g	5	89	186–189 [40]
8	4-NO2-Ph	4h	3	92	221–224 [37]
9	3-NO2-Ph	4i	3	90	225–227 [37]
10	3,4-(MeO)2-Ph	**4j**	5	86	173–175 [41]
11	4-OH-Ph	4k	5	87	254–257 [42]
12	4-OMe-Ph	4l	5	86	203–205 [37]
13	Thiophene	4m	5	88	215–217 [37]
14	4-(Me)2N-Ph	4n	5	88	250–253 [38]
15	4-F-Ph	4o	3	90	175–177 [43]
16	3-F-Ph	4p	5	92	209–211 [40]
17	2-F-Ph	4q	3	89	231–233 [39]
18	2,6-(Cl)2-Ph	**4r**	3	90	227–230 [38]

Reaction conditions: Benzaldehyde 1a–1r (1 mmol), ethyl acetoacetate 2 (1.5 mmol), urea 3 (2 mmol), and CoFe2O4@SiO _2_ -SO3H (0.015 g) with microwave irradiation at 400 W under solvent-free conditions.

Moreover, the 2-amino-4,6-diarylnicotinonitrile derivatives were synthesized using CoFe _2_ O _4_ @SiO _2_ -SO _3_ H MNPs under MW irradiation according to the method presented in a previous work [36] (Table 4).

**Table 4:** Four-component condensation of aldehydes, acetophenone, malononitrile, and ammonium acetate in the presence of CoFe _2_ O _4_ @SiO _2_ -SO _3_ H for the preparation of 2-amino-4,6-diarylnicotinonitrile.

No.	R	Product	Time (min)	Yield (%)	Mp (°C) Ref.
1	H	5a	2	89	187–189 [44]
2	4-Cl	5b	1.5	90	180–182 [44]
3	3-Cl	5c	2	87	168–170 [36]
4	2-Cl	5d	2	90	199–201 [45]
5	4-F	5e	1.5	92	164–166 [45]
6	3-F	5f	2	88	162–165 [36]
7	2-F	**5g**	2	85	178–180 [36]
8	4-NO2	5h	2	87	216–218 [45]
9	3-NO2	5i	2	88	208–210 [45]
10	4-Br	5j	1.5	92	186–188 [45]
11	4-CN	5k	1.5	92	185–187 [45]
12	2,6-(Cl)2	5l	2	86	174–176 [36]
13	2,4-(Cl)2	**5m**	1.5	89	179–181 [36]

Reaction conditions: Benzaldehyde 1a–1m (1 mmol), acetophenone 2 (1 mmol), malononitrile 3 (1 mmol), and ammonium acetate 4 (1 mmol) with 0.012 g CoFe2O4@SiO _2_ -SO3H under solvent-free conditions with microwave irradiation at 600 W.

In order to investigate catalyst reusability, after completion of the reaction, the catalyst was separated by an external magnet and then washed with ethanol. The CoFe _2_ O _4_ @SiO _2_ -SO _3_ H MNPs could be reused at least 5 times (Table 5).

**Table 5:** Reusability of the CoFe _2_ O _4_ @SiO _2_ -SO _3_ H nanocatalyst.

Run	Yield (%)
Fresh	90
First	90
Second	88
Third	88
Fourth	86

Reaction conditions: 4-Chlorobenzaldehyde 1 (1 mmol), urea 2 (2 mmol), ethyl acetoacetate 3 (1.5 mmol), and CoFe2O4@SiO _2_ -SO3H (0.015 g) with microwave irradiation at 600 W under solvent-free conditions.

Generally, employing the CoFe _2_ O _4_ @SiO _2_ -SO _3_ H MNPs gave an efficient method for the preparation of pure products in excellent yields.

### 2.3. Biological assessments

#### 2.3.1. Cytotoxicity

**Figure 5:**
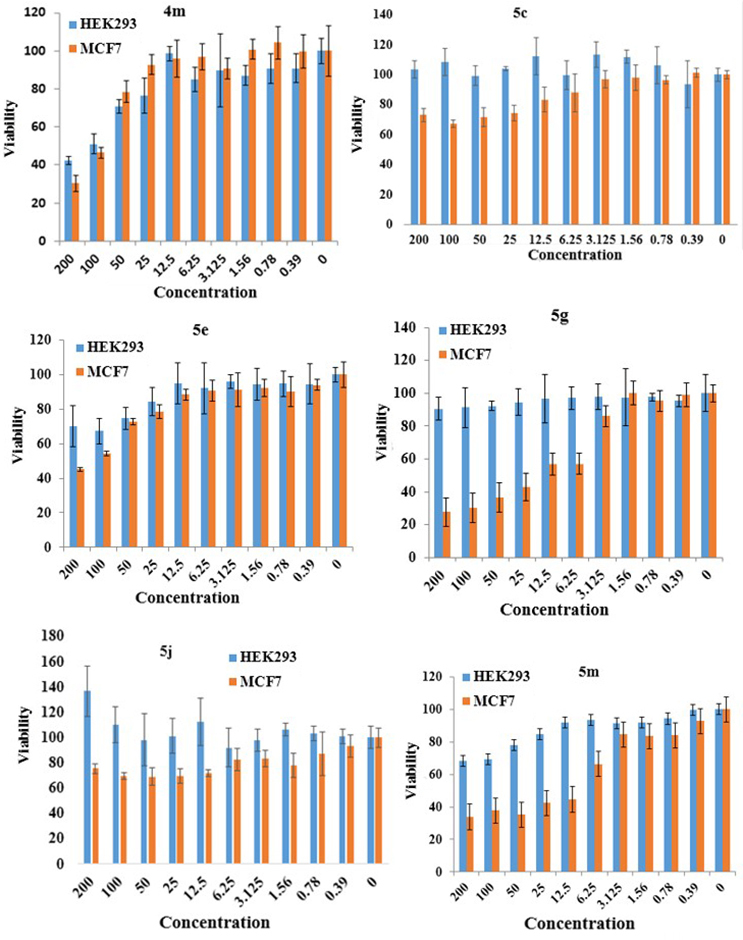
Treatment of AGS cells (24 and 48 h) with DHPMs derivatives ( **4j** and **4r** ) (200–1.56 μg/mL) and cell viability screening via MTT assay. Data are represented as mean ±SD (n = 3). *P <0.05, **P <0.01, ***P <0.001, ns: not significant.

Two human cancer model cell lines (MCF-7 and AGS) were treated with various concentrations (0.39–200 μg/mL) of pyrimidine and pyridine derivatives. Interestingly, the primary screenings exhibited high cytotoxic effects against AGS and MCF-7 cells with fewer side effects (low cytotoxic effects) on normal cells (HEK293). Cell viabilities were recorded after treatment for 24 and 48 h. As shown in Figure 5, after treatment for 24 and 48 h, compound **4r** induced death in ≥50% of AGS cells, while compound **4j** exhibited the effect after 48 h at different concentrations.

**Figure 6:**
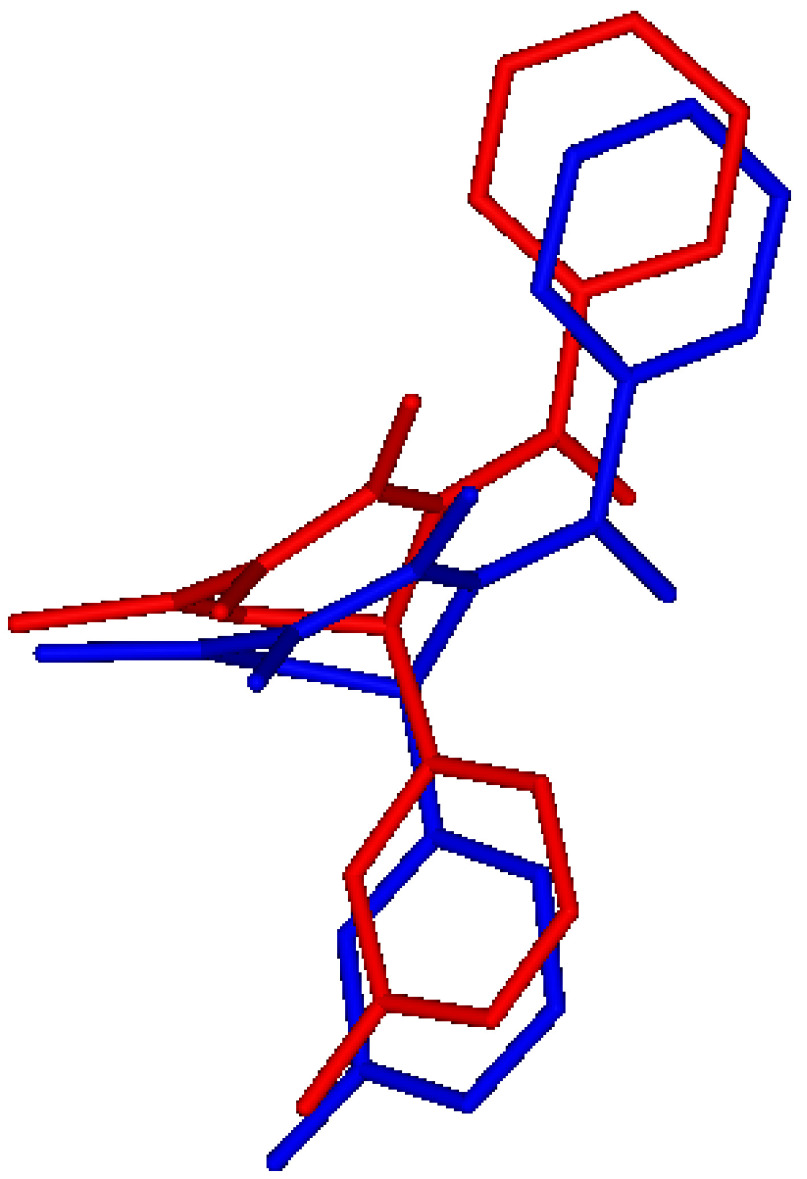
Treatment of MCF-7 and HEK293 cells (24 h) with compounds 4m, 5c, 5e, **5g** , 5j, and **5m** (0.39–200 μg/mL).

In the case of pyridine derivatives, **5g** and **5m** were less cytotoxic against HEK293 cells when compared to the other analogs. These compounds ( **5g** and **5m** ) demonstrated the highest effects, which led to a considerable decrease in cell viability in MCF-7 cells at different concentrations (Figure 6).

Among the compounds, compound **4r** exhibited the strongest cytotoxic effect towards the MCF-7 cell line, with an IC _50_ of 0.17 μg/mL. Moreover, compounds **4j** and **4r** showed superior cytotoxic activity against the AGS cell line after 48 h, with an IC _50_ of 4.90 and 4.97 μg/mL, respectively. Moreover, compounds **5g** , **5m** , and 4m were more potent against the MCF-7 cell line, with an IC _50_ of 71.94, 82, and 128 μg/mL, respectively. In contrast, compounds 5e (IC _50_ of 288.56 μg/mL), 5c (IC _50_ of 307 μg/mL), and 5j (IC _50_ of 615.88 μg/mL) were less toxic for the MCF-7 cell line (Tables 6 and 7). As shown in Table 7, it was found that, with the presence of an electronegative substituent at the ortho position, the cytotoxic activity had increased. In other words, in pyridine derivatives, the cytotoxic activity against the MCF-7 cell increased with the transfer of a substituent from para to ortho positions. Among the pyridine derivatives, the strongest and weakest cytotoxic effects belonged to **5g** (2-F derivative) and 5j (4-Br derivative), respectively. It was shown that the cytotoxic effect decreased by a greater amount with the para substituent in the brominated compounds.

**Table 6:** Cytotoxicity results for compounds **4j** and **4r** in the MTT assessment.

Entry	Compound	IC _50_ (μg/mL) AGS (24 h)	IC _50_ (μg/mL) AGS (48 h)	IC _50_ (μg/mL) MCF-7	IC _50_ (μg/mL) HEK293
1	**4j**	267.7	04.90	510	>200
2	**4r**	29.98	04.97	0.17	>200

**Table 7:** Cytotoxicity results of pyridine analogous in the MTT assessment.

Entry	Compound	IC _50_ (μg/mL) MCF-7	IC _50_ (μg/mL) HEK293
1	4m	128.00	146.25
2	5c	307.00	>1000
3	5e	288.56	157.24
4	**5g**	71.94	949.59
5	5j	615.88	195.00
6	**5m**	82.90	258.67

### 2.4. Molecular docking

Recent drug discovery methods have relied heavily on the crystallographic 3D structural data of biomolecular compounds. Numerous crystallographic records in the Protein Data Bank (PDB) have facilitated the effectiveness of molecular modeling research aiming at Eg5 as a validated target for cancer. One of the most popular molecular modeling strategies is docking, which provides a technique to simulate the stereoelectronic fitness of receptors and ligands via the lowest energy pathway.

#### 2.4.1. Docking validation

**Figure 7:**
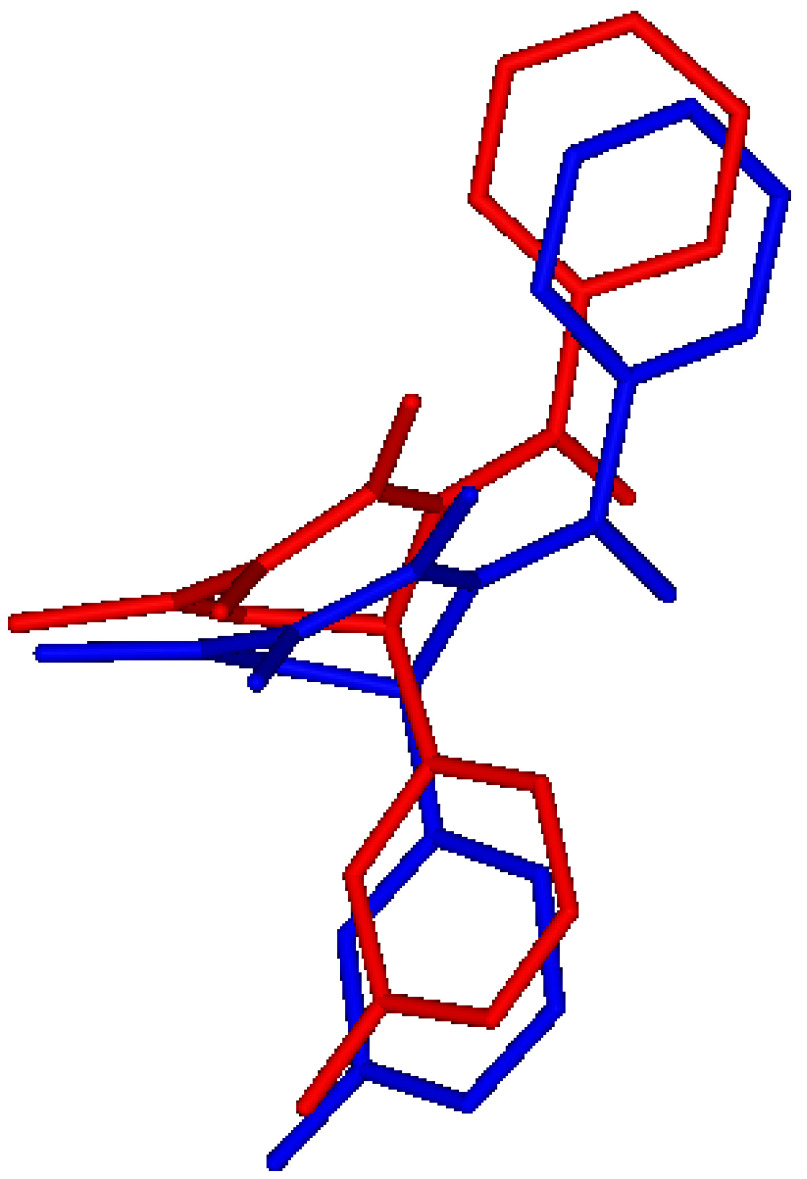
Binding poses of MOY (cognate ligand) within the Eg5 active site (PDB ID: 2IEH) in docked blue stick and crystallographic red stick states (RMSD 0.61 ?).

Accuracy of a typical docking protocol could be examined by self-docking measurement of the root mean square deviation (RMSD) in the docked and crystallographic poses. The docking validation method was conducted for 11 PDB structures of Eg5. Enzyme conformations were selected based on consideration of the relative similarity of the cocrystallized ligands to the structures of the DHPM and the crystallographic resolutions. Regarding the RMSD values and conformation population in the top-ranked cluster of an AutoDock output file, the enzyme structure with PDB code 2IEH was selected as the most appropriate crystallographic structure for further modeling studies. Docking validation results are illustrated in Table 8. Moreover, Figure 7 shows the docked MOY and the cocrystallized one in almost the same positions among the receptors (RMSD = 0.61 ?) that confirmed validation of the docking protocol.

**Table 8:** Results of docking validation for various holo PDB structures of Eg5 with 50 GA runs and 2.5 ×10 ^6^ maximum number of energy evaluations.

PDB code	Resolution (Å)	Population in the optimum cluster (%)	RMSD from references structure (Å)
3K3B	2.40	50	0.355
2X2R	2.20	23	0.610
2IEH	2.70	50	0.610
2X7D	2.30	50	0.850
2X7C	1.90	48	0.740
4A51	2.70	19	0.779
3KEN	2.50	48	0.814
4BBG	2.75	31	0.892
2X7E	2.40	50	0.897
2XAE	2.60	32	0.931
4A50	2.75	48	1.951

#### 2.4.2. Docking analysis

**Figure 8:**
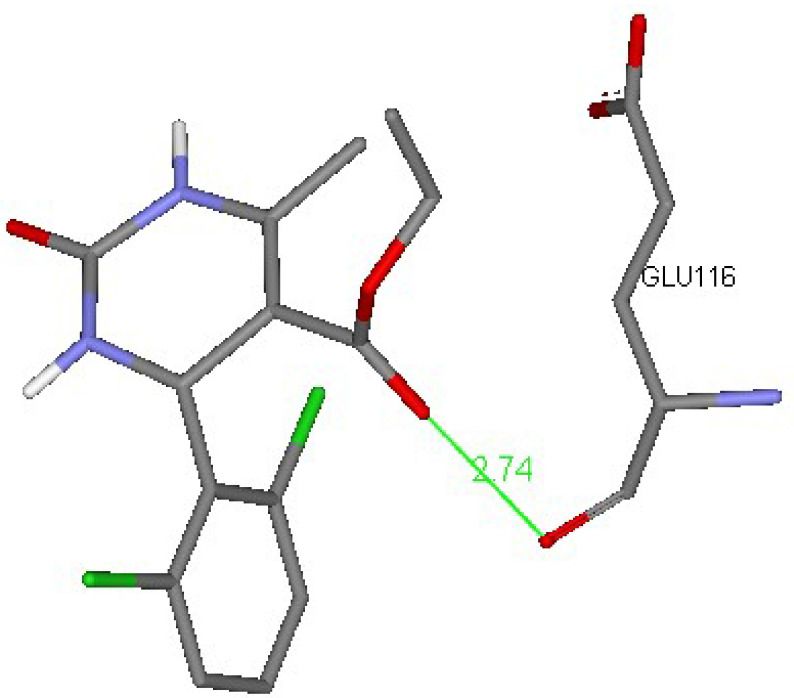
3D interaction schemes of a docked ligand ( **4r** ) in the Eg5 active site (PDB ID: 2IEH).

**Figure 9:**
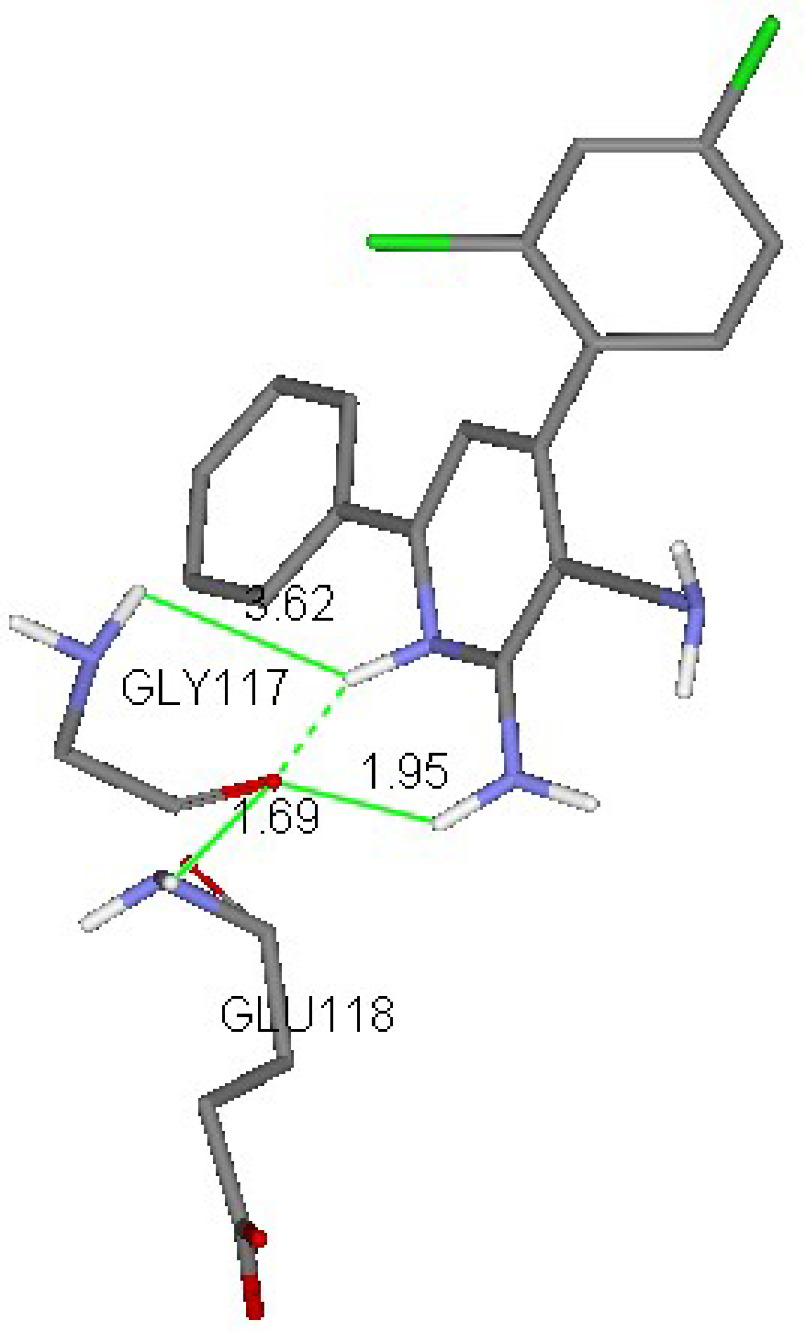
3D interaction scheme of a docked ligand ( **5m** ) in the Eg5 active site (PDB ID: 2IEH).

The estimated free binding energies, the populations in the optimum clusters, and the calculated inhibition constants (ki) of the synthesized compounds extracted from the docking studies are summarized in Table 9, where it can be seen that compounds **4r** and **5m** showed the highest free energies of binding, which indicated their probable binding ability to Eg5 residues. These products had the highest values in their groups, because there were 2 kinds of molecules. Structure binding explorations proved that H-bonds and hydrophobic contacts were responsible for such interactions. Compound **4r** interacted with Glu116 to ensure the stability of the α-helix [46]. Compound **5m** exhibited hydrogen bonds with Glu118 and Gly117 (Figures 8 and 9).

**Table 9:** Results of docked DHPs and pyridine derivatives within the Eg5 active site (PDB code: 2IEH).

Compound	Population in the optimum clusters (%)	Estimated Ki (μM)	Estimated free energy of binding (Kcal/mol)
**4j**	14	13.00	–6.67
4m	28	30.97	–6.15
**4r**	13	02.39	–7.67
5c	42	0.352	–8.80
5e	24	0.941	–8.22
**5g**	50	0.764	–8.24
5j	50	0.312	–8.88
**5m**	50	0.105	–9.52

**Figure 10:**
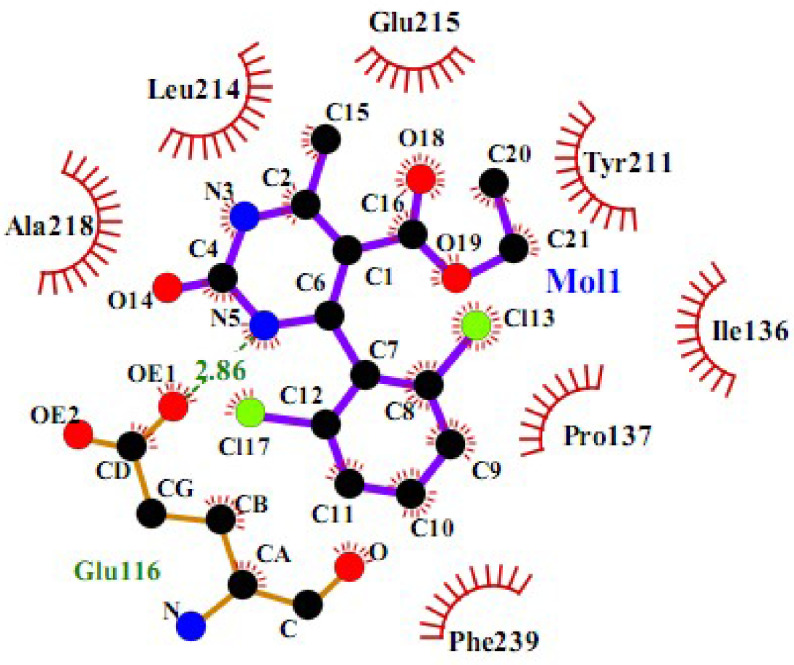
2D interaction scheme of a DHPM derivative ( **4r** ) in the Eg5 active site (PDB ID: 2IEH).

**Figure 11:**
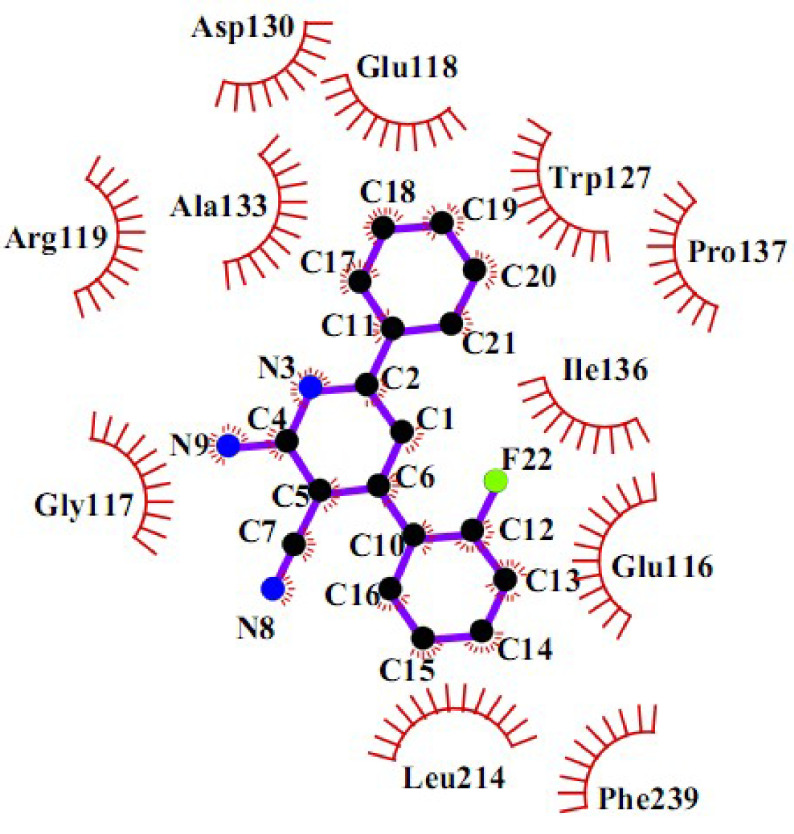
2D interaction scheme of a pyridine derivative ( **5g** ) in the Eg5 active site (PDB ID: 2IEH).

**Figure 12:**
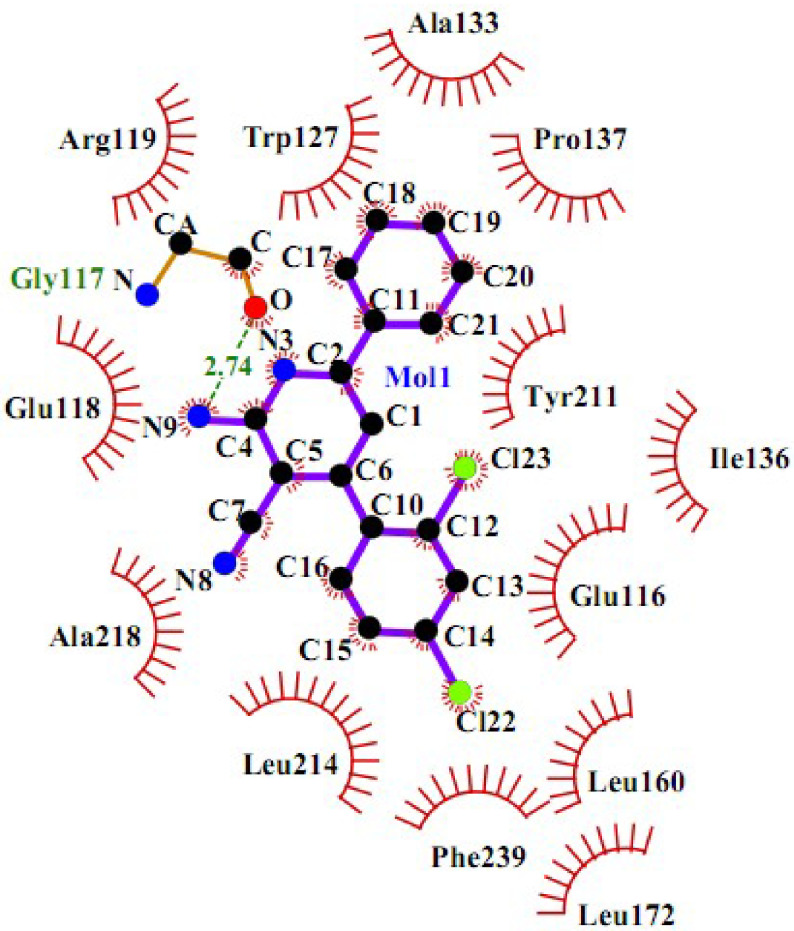
2D interaction scheme of a pyridine derivative ( **5m** ) in Eg5 active site (PDB ID: 2IEH).

To get more data, a 2D schematic presentation of the ligand–receptor interactions for the 3 compounds, with high energy and good cytotoxic activity, is displayed in Figures 10–12. According to the schematic binding representations, H-bond interaction could also be detected between the Glu116 and NH (ring) of compound **4r** , whereas H-bond donor interaction between Glu118 and NH (ring) was recorded for compound **5m** . The docking analysis revealed that all of the compounds interacted with Eg5 in an appropriate manner, with the dominance of a H-bond donor group in the DHPM ring.

In past reports, the existence of some drawbacks, such as harsh conditions, toxic organic solvents, long reaction times, high costs, difficult reuse, low product yields, and eco-unfriendly catalysts have limited the use of these methods [19–26]. Therefore, this paper reports a novel, simple, and efficient strategy for the preparation of pyrimidine and pyridine derivatives with anticarcinogenic potential, without solvent, under microwave irradiation, and using magnetic cobalt ferrite nanoparticles as the catalyst. Based on the literature, thio-derivatives of monastrol (pyrimidine scaffold) have displayed relevant antiproliferative effects against melanoma (UACC.62), kidney (786-0), breast (MCF-7), ovarian (OVCAR03), and particularly colon (HT-29) cancer cell lines [5], while the results herein strongly suggest that DHPMs inhibit important tumorigenic features of AGS cancer cells, leading them to death. Among the pyridine derivatives, a series of 2-amino-3-cyano-6-(1H-indol-3-yl)-4-phenyl pyridine derivatives have shown cytotoxic activity against four human cell lines (A549, H460, HT-29, and SMMC-7721) that form preliminary structure–activity relationships, and the introduction of halogen groups into the benzene ring was essential for their cytotoxic activity [11]. To the best of our knowledge, the cytotoxic assessment of five derivatives, 5c, 5f, **5g** , 5l, and **5m** , has not been previously reported, and among them, compounds **5g** and **5m** exhibited good cytotoxic activity against the MCF-7 cell line. Affinity maps of the ligand–enzyme showed that derivatives with halogen groups in the benzene ring had the potentiality to interact with the Eg5 binding site via H-bond interactions. Based on the reports and results herein, hydrogen bond formation and hydrophobic interactions were the key factors affecting the inhibitory effect of these compounds [1].

## 3. Experimental

### 3.1. Chemicals

All chemical materials, solvents, salts, and other chemical materials were commercially purchased from Sigma- Aldrich (St. Louis, MO, USA), Fluka (Dorset, England, UK), and Merck (Darmstadt, Germany). Thin-layer chromatography (TLC) was used to follow the reactions.

### 3.2. Synthesis

#### 3.2.1. Synthesis of CoFe _2_ O _4_ @SiO _2_ -SO _3_ H

This catalyst was prepared in three steps according to method presented in a previous report [36].

#### 3.2.2. Experimental procedure for the synthesis of DHPM and 2-amino-4,6-diarylnicotinonitrile derivatives

After characterization of the catalyst, its catalytic effect on the formation of the DHPMs and 2-amino-4,6-diarylnicotinonitrile derivatives was evaluated.

In the first step, in order to optimize the reaction conditions, the reaction of 4-chlorobenzaldehyde, ethyl acetoacetate, and urea under different microwave powers in the absence and presence of various amounts of CoFe _2_ O _4_ @silica sulfuric acid were performed as a model reaction (Tables 1 and 2). The progress of the reaction was monitored via TLC after every minute (EtOAc and n-hexane, 6:10). After completion of the reaction, the solid product was filtered and the pure product was obtained by recrystallization from hot ethanol. The CoFe _2_ O _4_ @SiO _2_ -SO _3_ H nanocatalyst was subsequently washed with ethanol and then dried for use in the next catalytic cycles.

For optimization of the catalyst amount, different quantities of catalyst were evaluated. As can be seen in Table 1, in the absence of a nanocatalyst, only trace amounts of the products could be achieved. When the reaction was performed in the presence of 0.015 g of the nanocatalyst, an excellent yield of products was achieved (Table 1, entry 3). Finally, 0.015 g of the nanocatalyst and 400 W of microwave irradiation under solvent-free conditions were obtained as the optimum conditions.

After optimization of the reaction conditions, reactions of ethyl acetoacetate with a wide range of substituted aldehydes (1a–1r) and urea were performed in the presence of CoFe _2_ O _4_ @silica sulfuric acid MNPs under solvent-free conditions with microwave irradiation within 3–5 min (Table 3). The yields of all of the reactions were good to excellent. It was found that aldehydes with electron-donating groups represented lower reactivity when compared with aldehydes with electron-withdrawing groups.

Next, the 2-amino-4,6-diarylnicotinonitrile derivatives were synthesized using CoFe _2_ O _4_ @SiO _2_ -SO _3_ H as a nanocatalyst under microwave radiation according to the method presented in a previous project [36]. In a 5-mL microwave reaction vessel, a mixture of ammonium acetate 4 (1.5 mmol), malononitrile 3 (1 mmol), acetophenone 2 (1 mmol), an aromatic aldehyde (1a–1m) (1 mmol), and CoFe _2_ O _4_ @silica sulfuric acid (0.012 g) was reacted for 1–2 min. The reaction progress was elucidated via TLC after every minute (n-hexane and EtOAc, 10:6). It was found that aldehydes with electron-withdrawing groups in the ortho and meta positions reacted slower than those in the para position. This catalyst was separated from the mixture using an external magnet and washed for the recycling tests. All of the products were known compounds that were characterized by melting point, IR, and ^1^ H NMR and ^13^ C NMR spectra.

### 3.3. Structural characterization

Proton NMR spectra were recorded on a Bruker DRX-250 Avance spectrometer (Billerica, MA, USA) at 250.13 MHz. Proton chemical shifts were reported in ppm (δ) relative to the internal standard tetramethylsilane (TMS, δ 0.0 ppm) or with the solvent reference relative to the TMS employed as an internal standard (CDCl _3_ , δ 7.26 ppm). Carbon NMR spectra were recorded on a Bruker DRX-250 Avance spectrometer at 62.90 MHz with complete proton decoupling. Carbon chemical shifts were reported in ppm (δ) relative to TMS with the respective solvent resonance as the internal standard (CDCl _3_ , δ 77.0 ppm). The probe temperature was regulated at 27 °C. For each spectrum, 64 scans were collected using 30°pulses with a spectral width of 20.5 ppm, acquisition time of 2.67 s, and recycle delay of 3 s. IR spectra were recorded on KBr discs on a Jasco 6300 FTIR spectrometer (Tokyo, Japan). The microwave-assisted approaches were performed in a Milestone microwave oven (Sorisole, Italy) operating at 1600 W. Melting points were recorded on an Electrothermal 9100 apparatus (LABEQUIP Ltd., Markham, Canada). The structural characteristics of CoFe _2_ O _4_ @SiO _2_ -SO _3_ H MNPs were studied via XRD with an X’Pert-PRO advanced diffractometer (Malvern Instruments, Malvern, UK) using Cu (Ka) radiation (wavelength: 1.5406 Å). The morphologies and sizes of the particles were studied using a scanning electron microscope (KYKY Co., Beijing, China, model: EM 3200).

### 3.4. Spectral data of some products

#### 3.4.1. 2-Amino-4-(3-chlorophenyl)-6-phenylnicotinonitrile (5c)

Yellow crystal, yield: 87%, mp 168–170 °C; IR (KBr): 3469 and 3305 (NH2) , 2205 (CN), 1635 (C=N), 1578 (C=C), 1258 (C-N); ^1^ H NMR (250.13 MHz, DMSO): δ 7.06 (s, 2H, NH2) , 7.30 (s, 1H, aromatic), 7.46–7.61 (m, 6H, aromatic), 7.74 (s, 1H, aromatic), 8.12 (d, 2H, aromatic, *J* = 3.5 Hz); ^13^ C NMR (62.90 MHz, CDCl _3_ ) : δ 110.99 (pyridine C-5), 116.68 (CN), 126.38, 127.32, 128.22, 128.81, 129.84, 130.20, 130.35, 134.90, 137.66, 138.61 (pyridine C-4), 153.51 (pyridine C-6), 160.17 (pyridine C-2) [36].

#### 3.4.2. 2-Amino-4-(3-fluoroophenyl)-6-phenylnicotinonitrile (5f)

Cream solid, yield: 88%, mp 162–165 °C; IR (KBr): 3473 and 3311 (NH2) , 2206 (CN), 1645 (C=N), 1575 (C=C), 1234 (C-N); ^1^ H NMR (250.13 MHz, DMSO): δH 7.03 (s, 2H, NH2) , 7.25 (s, 1H, aromatic), 7.34–7.47 (m, 5H, aromatic), 7.72 (t, 2H, aromatic, *J* = 5.5 Hz), 8.10 (d, 2H, aromatic, *J* = 5 Hz); ^13^ C NMR (62.90 MHz, CDCl _3_ ) : δC 111.08 (pyridine C-5), 115.89 (d, ^2^ J _CF_ = 22.01 Hz), 116.24 (CN), 116.99, 127.32, 128.80, 130.08 (d, 3JCF = 08.80 Hz), 133.00, 137.82, 154.00 (pyridine C-4), 160.25 (d, ^1^ J _CF_ = 250.97 Hz), 161.69 (pyridine C-6), 165.68 (pyridine C-2) [36].

#### 3.4.3. 2-Amino-4-(2,6-dichlorophenyl)-6-phenylnicotinonitrile (5l)

Yellow crystal, yield: 86%, mp 174–176 °C; IR (KBr): 3489 and 3373 (NH2) , 2214 (CN), 1666 (C=N), 1577(C=C), 1215 (C-N); ^1^ H NMR (250.13 MHz, DMSO): δ 7.18 (s, 2H, NH2) , 7.26 (s, 1H, aromatic), 7.46–7.57 (m, 4H, aromatic), 7.65–7.68 (m, 2H, aromatic), 8.09 (d, 2H, aromatic, *J* = 2.5 Hz); ^13^ C NMR (62.90 MHz, CDCl _3_ ) : δ 111.80 (pyridine C-5), 115.56 (CN), 127.39, 128.39, 128.79, 130.35, 130.84, 134.05, 137.63, 150.77 (pyridine C-4), 159.61 (pyridine C-6), 160.22 (pyridine C-2) [36].

#### 3.4.4. 2-Amino-4-(2,4-dichlorophenyl)-6-phenylnicotinonitrile ( **5m** )

Dark yellow crystal, yield: 89%, mp 179–181 °C; IR (KBr): 3480 and 3377 (NH2) , 2212 (CN), 1682 (C=N), 1615 (C=C), 1266 (C-N); ^1^ H NMR (250.13 MHz, DMSO): δ 7.13 (s, 2H, NH2) , 7.21 (s, 1H, aromatic), 7.45–7.47 (m, 3H, aromatic), 7.55–7.58 (m, 3H, aromatic), 8.09 (d, 2H, aromatic, *J* = 5 Hz); ^13^ C NMR (62.90 MHz, CDCl _3_ ) : δ 111.98 (pyridine C-5), 116.05 (CN), 127.38, 127.55, 128.84, 130.16, 131.14, 133.23, 134.36, 136.11, 137.59, 151.87 (pyridine C-6), 159.87 (pyridine C-2) [36].

### 3.5. Cytotoxicity studies

FBS, PBS, trypsin, penicillin, streptomycin, DMSO, 3-[4,5-dimethyl thiazol-2-yl]-2,5-diphenyltetrazoliumbromide (MTT), Triton X-100, and RPMI-1640 medium supplemented with 10% heat inactivated FBS were purchased from Sigma-Aldrich. The AGS, HEK293, and MCF7 cell lines were purchased from the National Cell Bank of Iran, Pasteur Institute of Iran, Tehran, Iran.

#### 3.5.1. Cell culture and cell proliferation assay via MTT

In this research, the effects of synthesized compounds on the viability of MCF-7, AGS, and HEK cells were evaluated at various concentrations (0.39–200 μg/mL) utilizing 3-(4,5-dimethylthiazol-2-yl)-2,5-diphenyltetrazolium bromide (MTT) assay [47]. After thawing, the cells were cultured in RPMI 1640 medium containing 10% FBS, penicillin (100 U/mL), and streptomycin (100 mg/mL) at 37 °C in a humidified 5% CO2 incubator. The cells were seeded in a 96-well plate at a density of 5000 cells per well (the cells were stained with trypan blue and counted with a hemocytometer). The cells were incubated overnight at 37 °C before cell viability testing. A stock suspension of each of the synthesized compounds at 50 mg/mL in distilled water was prepared. After that, fresh suspensions of different concentrations of the synthesized compounds (2-fold serial dilutions from 0.39 to 200 μg/mL) were made using serial dilutions of the stock suspensions of the synthesized compounds in the RPMI 1640 medium immediately before use. Next, 200 μL of suspension (different concentrations of synthesized compounds) was added to each well of the micropentaplicates. The cells were incubated for 24 and 48 h under the same conditions. Wells without any synthesized compounds served as a negative control. The experiments were performed in pentaplicate for each concentration. To assess cell survival, 100 μL of MTT solution (2 mg/mL in PBS) was added to each well and incubated for 3 h at 37 °C to produce insoluble formazan. Next, 100 μL of DMSO was added to dissolve the formazan crystals, and the absorbance was measured using an Infinite M200 ELISA plate reader (Tecan, Männedorf, Switzerland) at 570 nm, with 630 nm as a reference wave length. The percentage of cell viability was calculated via the formula (A _test_ /A _control_ ) ×100, in which A _test_ is the average absorbance of the treated cells and A _control_ is the average absorbance of the negative control [47].

### 3.6. Molecular docking

ChemDraw Ultra 8 (2D), ChemDraw 3D, AutoDock 4.2 (ADT1.5.4), Accelrys Viewer Lite 5.0, LigPlot, and Notepad++ were used.

#### 3.6.1. Study of molecular docking

Studies of flexible-ligand docking were performed using AutoDock version 4.2. The PDB was employed to retrieve all holo crystallographic structures of Eg5 with PDB codes 3K3B [48], 2X2R [49], 2XAE [49], 2IEH [50], 2X7D [51], 2X7C [51], 2X7E [51], 4A51 [52], AND4A50 [52], and 3KEN [53] in this work.

All crystallographic water molecules and cognate ligands (MOY) were eliminated from the original receptor structure for the provision of a target protein as a model (PDB code: 2IEH). All of the procedures for the target/ligand preparation were conducted according to previous reports [54]. LIGPLOT was used for the schematic 2D representations of the ligand-receptor interactions [55].

### 3.7. Analysis of statistical data

Cytotoxicity assessment of the cell lines were performed in pentaplicate, and the results were computed as mean ±SD. The experimental results were analyzed using one-way ANOVA analysis in SPSS 16.0 for Windows (SPSS Inc., Chicago, IL, USA) and P <0.05 was considered significant. The IC _50_ values were calculated by nonlinear regression analysis using Finney probit analysis in SPSS.

### 3.8. Conclusions

In the present work, a very facile and efficient method was reported for the synthesis of pyrimidine and pyridine derivatives in the presence of CoFe2 @SiO _2_ -SO _3_ H as a recoverable catalyst under microwave irradiation and solvent-free conditions. The results revealed that the synthesized DHPM derivatives had the potentiality to bind to the Eg5 binding site ( **4r** and **5m** ) via H-bond interactions and hydrophobic contacts with GLU116, GLU118, and GLY117 residues. Biological assessments showed that the candidate molecules exhibited appropriate cytotoxic effects against MCF-7 and AGS cells with low cytotoxicity to normal embryonic kidney (HEK293) cells. Such advantages afforded the great potential of these compounds to be modified and developed towards more selective anticancer agents.

## References

[1] (2013). Synthesis and molecular modeling of six novel monastrol analogues: evaluation of cytotoxicity and kinesin inhibitory activity against HeLa cell line. DARU Journal of Pharmaceutical Sciences.

[2] (2018). Granule-dependent NK cell killing of cryptococcus requires kinesin to reposition the cytolytic machinery for directed cytotoxicity. Cell Reports.

[3] (2018). Identification of novel scaffolds to inhibit human mitotic kinesin Eg5 targeting the second allosteric binding site using in silico methods. Journal of Receptors and Signal Transduction.

[4] (2011). Synthesis of dihydropyrimidin-2-one/thione library and cytotoxic activity against the human U138 MG and Rat C6 glioma cell lines. Journal of the Brazilian Chemical Society.

[5] (2006). Synthesis and differential antiproliferative activity of Biginelli compounds against cancer cell lines: monastrol, oxo-monastrol and oxygenated analogues. Bioorganic Chemistry.

[6] (2014). Synthesis and in vitro and in vivo antitumor/anticancer activity of novel O-Mannich bases of 4, 6-diaryl-. Journal of the Iranian Chemical Society.

[7] (2007). Synthesis and pharmacological studies of some 6-aminopyrimidin-4-ones. Indian Journal of Heterocyclic Chemistry.

[8] (2009). Synthesis and pharmacological investigation of 3-(substituted 1-phenylethanone)-4-(substituted phenyl. European Journal of Medicinal Chemistry.

[9] (2012). Evaluation of the in vitro antiretroviral potential of some Biginelli-type pyrimidines. Acta Virologica.

[10] (2019). -thiones as Eg5 inhibitors and L-type calcium channel blockers: potential antitumor dual agents. Medicinal Chemistry Communication.

[11] (2011). Synthesis and antitumor activity of 2-amino-3-cyano-6-(1H-indol-3-yl)-4-phenylpyridine derivatives in vitro. European Journal of Medicinal Chemistry.

[12] (2007). Discovery of structurally diverse HIV-1 integrase inhibitors based on a chalcone pharmacophore. Bioorganic Medicinal Chemistry.

[13] (2011). Synthesis and anti-microbial activity of some 1- substituted amino-4,6-dimethyl-2-oxo-pyridine-3-carbonitrile derivatives. European Journal of Medicinal Chemistry.

[14] (2003). Discovery of novel and selective IKK-$\beta $ serine-threonine protein kinase inhibitors. Part 1. Bioorganic {\&} Medicinal Chemistry Letters.

[15] (2005). Novel milrinone analogs of pyridine-3-carbonitrile derivatives as promising cardiotonic agents. European Journal of Medicinal Chemistry.

[16] (2008). -substituted nicotinonitriles as A2A adenosine receptor antagonists. Journal of Medicinal Chemistry.

[17] (2001). Possibility of the reversal of multidrug resistance and the avoidance of side effects by liposomes modified with MRK-16, a monoclonal antibody to p-glycoprotein. Journal of Controlled Release.

[18] (2008). Cancer multidrug resistance (MDR): a major impediment to effective chemotherapy. Asian Pacific Journal of Cancer Prevention.

[19] (2016). New efficient synthesis of 3, 4-dihydropyrimidin-. Molecules.

[20] (2015). Catalytic performance of bismuth pyromanganate nanocatalyst for Biginelli reactions. RSC Advances.

[21] (2015). Ultrasound assisted one-pot synthesis of dihydropyrimidinones using holmium chloride as catalyst. Journal of Sciences of the Islamic Republic of Iran.

[22] (2015). An efficient synthesis of 3, 4-dihydropyrimidin-. Molecules.

[23] (2010). Synthesis of some new 2-amino-3-cyano-4-aryl-6-(1-naphthylamino)-pyridines as antibacterial agent. Journal of Chemical and Pharmaceutical Research.

[24] (2009). An efficient one-pot synthesis and in vitro antimicrobial activity of new pyridine derivatives bearing the tetrazoloquinoline nucleus. Arkivoc.

[25] (2009). Synthesis and antimicrobial activity of some new cyanopyridine and cyanopyrans towards \textit{Mycobacterium tuberculosis} and other microorganisms. Indian Journal of Chemistry.

[26] (2007). Synthesis and evaluation of antifungal properties of a series of the novel 2. -phenyl-5.

[27] (2010). Three-component reaction of isocyanides and 2-formylbenzoic acid with dibenzylamine catalyzed by silica nanoparticles under solvent-free conditions. Helvetica Chimica Acta.

[28] (2011). Silica nanoparticles as a highly efficient catalyst for the one-pot synthesis of 2-hydroxyacetamide derivatives from isocyanides and electron-poor aromatic aldehydes. Helvetica Chimica Acta.

[29] (2010). Novel one-pot, four-component condensation reaction: an efficient approach for the synthesis of 2,5-disubstituted 1,3,4-oxadiazole derivatives by a Ugi-4CR/aza-Wittig sequence. Organic Letters.

[30] (2015). Synthesis of N-acylurea derivatives from carboxylic acids and. Journal of Chemical Sciences.

[31] (2011). Application of magnetic particles modified with amino groups to adsorb; copper ions in aqueous solution. Journal of Environmental Sciences.

[32] (2011). Amine-functionalized silica nanoparticle: Preparation, characterization and anionic dye removal ability. Desalination.

[33] (2011). -functionalized magnetic Fe$_{3}$O$_{4}$ nanoparticles as an efficient and reusable catalyst for one-pot synthesis of $\alpha $-amino nitriles in water. Applied Catalysis A: General.

[34] (2012). Nano-Fe$_{3}$O$_{4}$ encapsulated-silica particles bearing sulfonic acid groups as a magnetically separable catalyst for highly efficient Knoevenagel condensation and Michael addition reactions of aromatic aldehydes with 1,3-cyclic diketones. Chinese Journal of Catalysis.

[35] (2014). {4}$ nanoparticles as an efficient catalyst for the oxidation of alcohols to carbonyl compounds in the presence of oxone as an oxidant. Bulletin of the Korean Chemical Society.

[36] (2018). {3}$H as a reusable solid acid nanocatalyst under microwave irradiation in solvent-free conditions. Synthesis of 2-amino-4.

[37] (1H). Immobilization of phosphomolybdic acid nanoparticles on imidazole functionalized Fe$_{3}$O$_{4}$@SiO$_{2}$: a novel and reusable nanocatalyst for one-pot synthesis of Biginelli-type 3,4-dihydro-. RSC Advances.

[38] (2018). The role of pyruvic acid as starting material in some organic reactions in the presence of SBA-Pr-SO$_{3}$H nanocatalyst. Research on Chemical Intermediates.

[39] (2017). Preparation and characterization of ionic liquid functionalized SBA-15 and its application in the synthesis of 2, 3-dihydroquinazolinones. Iranian Journal of Catalysis.

[40] (2014). Synthesis of dihydropyrimidinones/thiopyrimidinones: Nafion-Ga, an efficient "green" Lewis acid catalyst for the Biginelli reaction. Catalysis Letters.

[41] (2016). New efficient synthesis of 3,4-dihydropyrimidin-. Molecules.

[42] (2015). Catalytic performance of bismuth pyromanganate nanocatalyst for Biginelli reactions. RSC Advances.

[43] (2011). Insights into conformational and packing features in a series of aryl substituted ethyl-6-. -oxo-1.

[44] (2009). A novel strategy for the synthesis of 2-amino-4,6-diarylnicotinonitrile. Arkivoc.

[45] (2016). Experimental and theoretical studies of the nanostructured \textbraceleft Fe$_{3}$O$_{4}$@SiO$_{2}$@(CH$_{2})_{3}$Im\textbraceright C(CN)$_{3\, }$catalyst for 2-amino-3-cyanopyridine preparation via an anomeric based oxidation. RSC Advances.

[46] (2015). -one derivatives on various breast cancer cell features. Impact of kinesin Eg5 inhibition by 3.

[47] (2016). In vitro antiplasmodial activity and cytotoxic effect of (Z)-2-benzylidene-. Iranian Journal of Parasitology.

[48] (2010). The discovery of tetrahydro-beta-carbolines as inhibitors of the kinesin Eg5. Bioorganic Medicinal Chemistry Letters.

[49] (2011). -$activity relationship and multidrug resistance study of new S-trityl-l-cysteine derivatives as inhibitors of Eg5. Journal of Medicinal Chemistry.

[50] (2007). Structure of human Eg5 in complex with a new monastrol-based inhibitor bound in the R configuration. Journal of Biological Chemistry.

[51] (2010). Structural basis for inhibition of Eg5 by dihydropyrimidines: stereoselectivity of antimitotic inhibitors enastron, dimethylenastron and fluorastrol. Journal of Medicinal Chemistry.

[52] (2012). Triphenylbutanamines: kinesin spindle protein inhibitors with in vivo antitumor activity. Journal of Medicinal Chemistry.

[53] (2010). Allosteric drug discrimination is coupled to mechanochemical changes in the kinesin-5 motor core. Journal of Biological Chemistry.

[54] (2014). -triazine derivatives: synthesis, cytotoxic activity and molecular docking studies on B-cell lymphoma 2 (Bcl-2). acenaphtho[1.

[55] (1995). a program to generate schematic diagrams of protein-ligand interactions. Protein Engineering, Design and Selection.

